# Development of Multilayer Microcapsules by a Phase Coacervation Method Based on Ionic Interactions for Textile Applications

**DOI:** 10.3390/pharmaceutics6020281

**Published:** 2014-06-13

**Authors:** Sudipta Chatterjee, Fabien Salaün, Christine Campagne

**Affiliations:** 1 University of Lille Nord de France, F-59000 Lille, France; E-Mail: sudipto_ch77@yahoo.co.in; 2 ENSAIT/GEMTEX, F-59100 Roubaix, France

**Keywords:** microcapsules, chitosan, oil in water emulsion, polyester fabric, air atmospheric plasma treatment, functional coating

## Abstract

The present study describes the development of multilayer microcapsules by 11 alternate additions of chitosan (Chi) and sodium dodecyl sulfate (SDS) in a combined emulsification and phase coacervation method based on ionic interactions. After an alkali treatment, microcapsules are applied on polyester (PET) fabric by a padding process to investigate their wash-durability on fabric. Air atmospheric plasma treatment is performed on PET fabric to modify the surface properties of the textiles. Zeta potential, X-ray photoelectron spectroscopy (XPS), wetting measurements, scanning electron microscopy (SEM), and atomic force microscopy (AFM) with surface roughness measurements are realized to characterize and determine wash durability of microcapsule samples onto PET. After alkali treatment, the microcapsules are selected for textile application because they are submicron sized with the desired morphology. The results obtained from various characterization techniques indicate that microcapsules are wash-durable on PET fabric pre activated by air plasma atmospheric as Chi based microcapsules can interact directly with PET by ionic interactions.

## 1. Introduction

Chitosan (Chi), a natural linear biopolymer of glucosamine monomers and small amount of *N*-acetyl-glucosamine monomers, is obtained by alkaline *N*-deacetylation of chitin, which is the second most abundant polysaccharide in nature after cellulose [1]. Chi is considered to be an attractive biomaterial in the area of microencapsulation technology due to its biocompatible, biodegradable and nontoxic nature [2]. Recently, Chi has received much attention for its applications in developing microcapsules, especially for its use as carriers for various intestine selective drugs by oral administration [3,4,5]. The main advantages of Chi-based microcapsules as drug carriers are their controlled release properties in a site-dependent manner and their biocompatibility [6,7]. Chi is also considered to be a good candidate for wall materials in food [8] and the encapsulation of textile finishing products [9]. Various methods such as spray drying and phase coacervation have been used for the formation of Chi microcapsules [10], and microcapsules are either single or multilayered, depending on the microencapsulation method [11].

The microencapsulation process is defined as an encapsulation of a compound into a membrane of polymers [12]. Nowadays, microcapsules are applied on textiles to impart different added values, such as long lasting fragrance, antimicrobial effect and thermal regulation. Health benefits by probiotic organisms are considered to be highly beneficial to the human body [13,14]. Moreover, different efforts are taken to improve the physical barrier and release properties for the encapsulation of various substances like drugs, nutrients, metals, acids, pesticides, herbicides, and flagrances [15,16]. Textile fabrics can provide a suitable substrate for the growth of microbes, especially at appropriate humidity and temperature in contact with the skin of the human body. After being fixed onto textile substrate, the loaded microcapsules with antimicrobial and anti-inflammatory agents can effectively interact with micro-organisms on skin [17] and also, microcapsules can show controlled release properties for various active agents loaded into it [18].

Chi shows interactions with anionic molecules and macromolecules such as natural or synthetic polymers and surfactants because each glucosamine unit possesses one functional amine (–NH_2_) group that is protonated in dilute aqueous acidic solutions [19,20]. The shell of Chi based microcapsules is obtained by electrostatic interaction between positively charged biopolymer and various negatively charged polyelectrolyte molecules like sodium alginate [21]. The electrostatic interaction between Chi and anionic microcapsules allows the formation of microcapsules in the emulsion system [22]. Microcapsules structure is stabilized by combination of different interactions such as electrostatic, ion-dipole, and hydrophobic [23]. Multifunctional properties of these microcapsules [24] are obtained due to their narrow particle size distribution, low diameter, wall permeability, and surface functionalization such as *N*-acylation [25] and *N*,*N*,*N*-trimethylation [26]. Chi particles are reported to be used for development of various carrier systems such as microspheres in lysozyme encapsulation [27], core shell morphology consisting of poly (*n*-butyl acrylate) core [28], and multilayer microcapsules for indomethacin microcrystals loading [29]. The advantages of multilayer microcapsules are based on the increase of the shell thickness and on the absence of a cross-linking step with the reactive agent.

The present study deals with the development of microcapsules by adding the required amount of Chi and sodium dodecyl sulfate (SDS) in order to control the deposition of wall materials. Eleven alternate additions of Chi and sodium dodecyl sulfate (SDS) were made to develop microcapsules in the oil in water emulsion system by combined emulsification and phase coacervation based on ionic interactions between Chi and SDS. The oil in water emulsion was developed using linseed oil as a model liposoluble phase. SDS was used as an anionic emulsifier. The formation of microcapsules by multilayer phase coacervation involved a rinsing step with deionized water. The rinsing step was first applied after three additions (Chi addition) prior to addition of SDS solution (four additions) in order to decrease viscosity of the microcapsules suspension and reduce repulsion between positively charged amine groups of Chi on the surface of microcapsules in the suspension. The rinsing step improved the dispersion of the system. The surface and morphology of microcapsules were examined using surface energy and atomic force microscopy (AFM). The alkali treatment of microcapsules was performed to solidify the outer microcapsule shells and improve their mechanical stability. The microcapsules obtained after alkali treatment were applied on woven polyester (PET) fabric by a padding process. For the textile applications, microcapsules formed by 11 alternate additions of SDS and Chi solution in the oil in water emulsion were chosen. The padding process (Pad-dry method) was applied to significantly improve the speed of microcapsules to PET fabric samples during washing and also to allow ionic interactions between positively charged amine (–NH_2_) groups of Chi in the microcapsules and carboxylic groups of PET fabric [30]. Atmospheric plasma treatment of fabric as preactivation of surface was applied to increase polar components on the surface of fabric and modify surface energy of PET fabric [31,32]. The wash durability of the microcapsules on the fabric was checked after fabric samples were subject to several washing cycles. The wash durability of microcapsules on the textile fabric was characterized by various techniques such as wetting measurement for the determination of contact angles and surface energies, zeta potential, X-ray photoelectron spectroscopy (XPS), scanning electron microscopy (SEM), and AFM.

## 2. Experimental Section

### 2.1. Materials

Low molecular weight Chi (molecular weight = 50,000–190,000 and deacetylation = 75%–85%), SDS, and linseed oil were provided by Sigma–Aldrich Co. LLC (Saint-Quentin Fallavier, France). The other analytical grade chemical reagents such as acetic acid, hydrochloric acid (HCl), sodium hydroxide (NaOH), diiodomethane were purchased from Sigma–Aldrich Co. LLC.

A woven polyester (PET) fabric obtained from Facotex Company (Roubaix, France) was used as textile substrate (weight of fabric 320 g m^−2^, thickness of 0.63 mm).

### 2.2. Preparation of Microcapsules

The formation of microcapsules by phase coacervation method based on ionic interactions involved alternate addition of SDS and Chi solution in the suspension at 50 °C and 1500 rpm. The microencapsulation process is based on the preparation of oil in water emulsion prior to phase coacervation. The oil in water emulsion was prepared by homogenizing (Ultra-Turrax, T-25 basic, Ika^®^werke, Staufen, Germany) 80 wt% of aqueous SDS (10 g·L^−1^) solution with 20 wt% linseed oil for 30 min at 16,000 rpm and 50 °C. The microcapsule formation was started by adding 15 mL (3%, *w*/*v*) of Chi solution in the oil in water emulsion under homogenization at 50 °C and 16,000 rpm for 10 min. Chi solution (3%, *w*/*v*) was prepared by completely dissolving 3.0 g of low molecular weight Chi powder in 100 mL aqueous solution of 2% (*v*/*v*) acetic acid at 1500 rpm and 50 °C. Microcapsule formation in the study was further followed by alternate SDS and Chi additions in the emulsion. Altogether, 11 alternate additions of SDS (20 mL) and Chi (10 mL for the first adding and 2.5 mL thereafter) solutions were made including rinsing steps with de-ionized water before each SDS addition. The rinsing step was first applied after three additions (Chi addition) prior to addition of SDS solution (four additions). The added volumes of Chi and SDS were based on the variation of the zeta potential value of the solutions during the process.

The alkali treatment of microcapsules was done to solidify the outermost shell of microcapsules. The alkali treatment step involved mixing of 5 mL of microcapsule suspension with 20 mL of 0.02 (N) NaOH at 30 °C and 1500 rpm for 10 min.

### 2.3. Sample Preparation for Textile Application

#### 2.3.1. Air Atmospheric Plasma Treatment of Woven Polyester (PET) Fabric

Plasma treatment of PET fabric was done under atmospheric pressure using the “Coating Star” plasma machine manufactured by Ahlbrandt System Company [33]. The plasma treatment was realized twice with an electrical power of 750 W, an electrical voltage of 15 kV, a frequency of 30 kHz, two successive electrodes of 1.5 cm width and 0.5 m length, and an electrode/counter T electrode gap of 1.5 mm and a treatment speed of 2 m·min^−1^.

#### 2.3.2. Pad-Dry Method

Two hundred milliliters of concentrated alkali treated microcapsules were applied on PET fabric at pH 5 with and without plasma pre-treatment by pad-dry method without using any binder. Chi solution was also fixed on PET fabric by pad-dry method. The curing was carried out at 50 °C for 30 min after the fixation.

#### 2.3.3. Washing Cycle Experiments of PET Fabrics Samples

Durability of microcapsules onto textile substrate was determined from washing tests of PET fabrics. Washing tests of the PET fabric samples were done with water in Washtech instrument (Roaches, Staffordshire, UK) at 40 °C for 30 min, followed by drying at 50 °C for 1 h. Microcapsules applied onto PET fabric for wash durability test included alkali treatment step.

### 2.4. Characterization

#### 2.4.1. Zeta Potential Measurement

The Zetasizer 2000, Malvern instruments Ltd., Malvern, UK was used to measure zeta potential of microcapsules suspensions by electrophoresis method. The zeta potential of the microcapsules sample during complex coacervation process was measured and the values were taken after diluting the samples in de-ionized water by 1000 times. The zeta potential measurement of microcapsules samples was done before alkali treatment.

The zeta potential of PET fabric samples was measured by streaming method using an electrolyte (KCl) solution in equipment provided by the company Zetacad [31]. The sample was introduced and maintained in the same KCl solution for 12 h in order to reach equilibrium before its measurement and the concentration of KCl was set at a concentration of 1.0 mM. The zeta potential of the fabric samples was measured at an initial pH of 4 in KCl solution and after each measurement the final pH of electrolytes was measured. The conductivity of the KCl solution was also determined after the measurement.

The zeta potential (ζ) of PET fabric samples was calculated by the following Equation [34]:

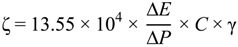
(1)
where ζ and *E* (voltage) are expressed in mV, *P* (pressure) in mbar and γ (solution conductivity) in S cm^–2^ and *C* is a constant term obtained from an empirical equation using device temperature (*T*) at 23 °C. The empirical equation is represented as follows:

*C* = 16.32 - 0.35197*T* + 0.00351*T^2^* (2)

Microcapsules applied on PET fabrics were obtained after alkali treatment.

#### 2.4.2. Wetting Measurement

The surface energy of solid samples (microcapsules and textile samples) was determined from contact angle values of sample with two different probe liquids by Owens and Wendt method [35]. The wetting measurement of microcapsules sample was done before alkali treatment of microcapsules but the characterization study regarding PET fabric with microcapsules included alkali treatment step. The contact angles of microcapsules samples in solid state were determined with GBX Digidrop Contact Angle meter by sessile drop technique. The Owens and Wendt method is based on the following equations:

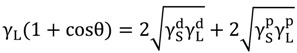
(3)

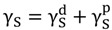
(4)
where θ is contact angle, and γ, γ^p^, and γ^d^ are total, polar component and dispersive component of surface energy, respectively. The two test liquids were water (γ = 72.8 mN m^−1^, γ^p^ = 51.0 mN m^−1^, and γ^d^ = 21.8 mN m^−1^) and diiodomethane (γ = 50.8 mN m^−1^, γ^p^ = 2.3 mN m^−1^, and γ^d^ = 48.5 mN m^−1^). The subscripts L and S denote liquid and solid, respectively. The samples of microcapsules for surface energy analysis were prepared by uniform deposition of solutions containing microcapsules on thin glass substrate, dried at room temperature conditions during 48 h.

The wettability of PET fabric samples was measured with water and diiodomethane (as liquid probes) on a “3S balance” from GBX Instruments (Bourg de Peage, France). Wetting force between the fabric samples and the solvent, and also the variation of the liquid solvent weight that went through the fabric length during a given time, were measured [33]. The latter parameter provided an estimation of the sample capillarity. During measurements, a rectangular-shaped fabric sample was connected to the “3S Balance” at the weighing position, and then progressively brought into contact with the solvent placed in a container which could move vertically up and down. As soon as the sample surface was in contact with the liquid solvent surface, a sudden increase in the sample weight was detected by the balance, which then automatically stopped the movement of the liquid container.

The contact angle of fabric samples with water and diiodomethane can be determined from the calculated meniscus weight using the following Equation [36]:

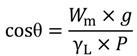
(5)
where *P* is perimeter of fabric in contact with liquid (mm), *W*_m_ is calculated meniscus weight (g), *g* is gravitational acceleration 9.81 m s^−2^, γ_L_ is surface tension of liquid (mN m^−1^), and θ (°) is the contact angle.

#### 2.4.3. Scanning Electron Microscopy (SEM) and Atomic Force Microscopy (AFM)

Scanning electron microscope (SEM) images of PET fabric samples were realized by Leica Cambridge S-360 Microscope (Leica Cambridge Instrument, Cambridge, UK) operated at an acceleration voltage of 20 kV. Microcapsules after alkali treatment were applied on PET fabric for characterization by SEM.

The surface roughness of microcapsule samples in solid state before alkali treatment was determined by atomic force microscopy (AFM) in ambient conditions using light tapping mode (TM-AFM), Nanoscope III digital instrument (version 3.2) equipped with an image processing software, version 3.2 (Digital Instrument Inc., Tonawanda, NY, USA). The set point frequency of silicon pyramidal cantilever with 4 to 6 Hz scan speed was about 272 Hz. The microcapsules samples used for AFM in dry state were prepared by making film on small and thin glass substrate. The mean roughness (*Ra*) of surface is expressed by the equation:

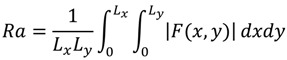
(6)
where *L_x_* and *L_y_* are the dimensions of surface, and *F*(*x*,*y*) is roughness curve relative to the center plane. The mean roughness is the average of *Ra* obtained at 10 different locations.

#### 2.4.4. Size Distribution Analysis by Granulometry

The granulometry analysis for size distribution of microcapsules was performed using Accusizer Particle Sizing Systems (770 Optical Particle Sizer, and 770A Autodiluter), Santa Barbara, CA, USA after diluting the microcapsules suspension by 1000 times in de-ionized water. The size distribution analyses of microcapsules samples were done before alkali treatment of microcapsules.

#### 2.4.5. X-ray Photoelectron Spectroscopy (XPS) of Textile Fabric Samples

PET fabric samples were analyzed by X-ray photoelectron spectroscopy (XPS) performed with ESCALAB 250 spectrometer (Thermo Scientific, Waltham, MA, USA) using a non-monochromatic Al *K*α standard (1486.6 eV) at a power of 300 W. The surface composition was obtained from the areas of the C1s, N1s and O1s peaks, obtained in the high resolution mode with pass energy: 40 eV for high resolution spectra and 100 eV for survey [26]. The C1s binding energy for the –C–C– groups was fixed to 285 eV and this value was taken as a standard for the binding energy calibration. The vacuum ~3.10–8 mbar was created during measurements. The dwell time during measurement was set at 50 ms for high-resolution spectra and 20 ms for survey. After alkali treatment, microcapsules were applied on PET fabric for XPS.

## 3. Results and Discussion

### 3.1. Formation of Multilayer Microcapsules in a Phase Coacervation Process

The formation of microcapsules by a phase coacervation process mainly involved electrostatic interactions between oppositely charged wall materials Chi and SDS [37] which led to shell membrane formation around oil droplets in the emulsion [23].

**Figure 1:**
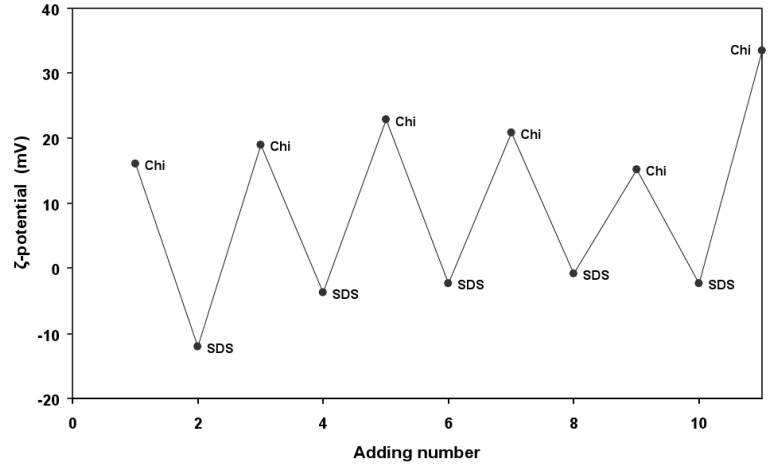
Zeta-potentials as a function of layer number during the layer by layer (LBL) multilayer deposition.

However, the amount of Chi adsorbed at the oil/water interface to form a monolayer is limited, and not sufficient to stabilize the particles during the recovering step. Therefore, the strategy was to use consecutive additions of SDS and Chi to increase the amount of polymeric material at the interface. The formation of microcapsules and/or polymeric films onto the particle surface was monitored by zeta potential measurement of the suspension after each addition of either Chi or SDS solution in the system; and plotted against the consecutive additions of SDS and Chi solutions in Figure 1. By following the proposed encapsulation method, oppositely charged polyelectrolyte and SDS were alternately deposited onto the surface until the desired adding numbers were reached. Figure 1 reflects the changes in the surface charge with depositions, which are negative and positive after SDS and Chi additions, respectively. The alteration of ζ-potential values due to the charge compensation indicated the successful deposition of each compound onto the surface. Furthermore, the absolute ζ-potential values of the couple Chi/SDS are relatively low, since Chi is a weak polyelectrolyte. It is interesting to note that the charge inversion is complete and quasi constant ζ-potential values around −3 and +20 mV are observed on SDS and Chi additions, respectively. When adsorbed on a Chi layer, part of the charge of SDS is compensated by the electrostatic interaction with Chi, and even if the remaining charge is not as high as the oil interface in the emulsion (−35 mV), it is sufficient to absorb the next coating layer of Chi.

In our previous work, we reported microcapsules (droplet particles) formation with four alternate layers of Chi and SDS. The zeta potential of microcapsules suspension was precisely controlled by adding suitable amount of SDS and Chi during formation of successive layers [38]. However, zeta potential of microcapsules suspension did not change to negative value after addition of SDS during formation of “layer 4” as SDS molecules failed to react with Chi molecules at the outermost surface of microcapsules due to incapability of Chi molecules to undergo conformational reorganization to maximize charge neutralization [38]. In this study, a rinsing step with de-ionized water was introduced after Chi addition (three additions) and before SDS addition (four additions). The same rinsing step was repeated during successive layer formation before SDS additions and it was mainly done to decrease viscosity of the microcapsules suspension and reduce repulsion between positively charged amine groups of Chi on the surface of microcapsules in the suspension.

**Figure 2:**
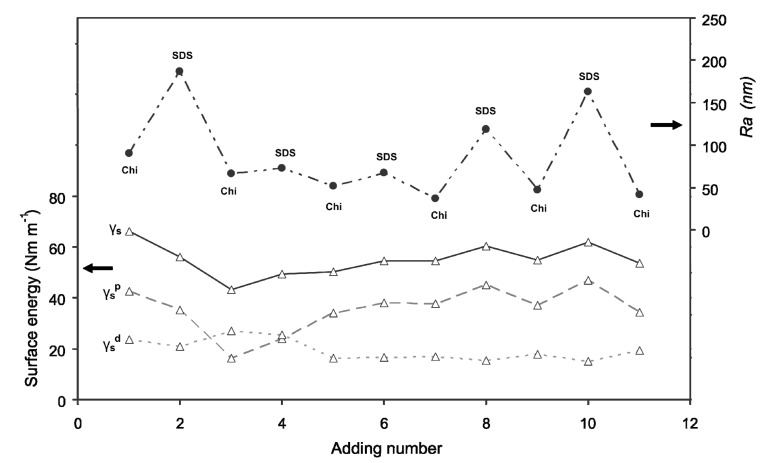
Polar component of surface energy (γ_s_^p^); dispersive component of surface energy (γ_s_^d^); and surface energy (γ_s_) of microcapsules samples; and roughness (*Ra*) as a function of layer number during the LBL multilayer deposition.

To obtain a rough estimation of the surface of the particles, atomic force microscopy (AFM) was performed on capsules during the deposition sequence. The *Ra* values (Figure 2) of microcapsules reveal that SDS addition leads to the formation of a rougher surface than Chi addition. Chi not only deposited on the microcapsules surface, but also wrapped around the micelles formed by SDS molecules [39], which resulted in a decrease of the polar component of the mixture (Figure 2). At this stage, some aggregations of the particles might occur due to precipitation of the absorbed and free dissolved Chi at pH 3.5 in the system [40]. The microcapsules become negatively charged after SDS addition because of their deposition on the surface of microcapsules. Nevertheless, this leads to the formation of intermolecular aggregates involving several Chi chains bridged by SDS micelles and this aggregation may be a consequence of the stiffness of the Chi chains. After being subject to rinsing step after three additions, the polar component of the surface energy increases as the number of additions is increased up to right, and that underlines the migration of polar groups on the surface. The increase in polar component value of surface energy more likely indicates the formation of well-defined particles in the system. The increase in polar component of surface energy and the subsequent increase in the hydrophilic nature of microcapsules could be due to tail-to-tail rearrangement of SDS molecules at the interface. From eight additions, the surface energy and more specially its polar component of the particles vary between 35 and 47 mN m^−1^. The Chi addition (e.g., 9 and 11 additions) results in a decrease in polar component of surface energy, since its cationic sites are binding with the anionic groups of SDS, and therefore particles present more hydrophobic surface. As shown in Figure 2, the surface roughness (*Ra*) does not vary a lot, and the surface is relatively smooth between four to seven additions. However, the samples after SDS additions are found to be rougher than that of the Chi additions. The increase in surface roughness with SDS additions influences the contact angle value, which tends to decrease. Thereby, the characterization of microcapsules using wetting experiments and AFM could be a great help to understand the microencapsulation process applied in textile fields for possible bio-medical applications.

**Figure 3:**
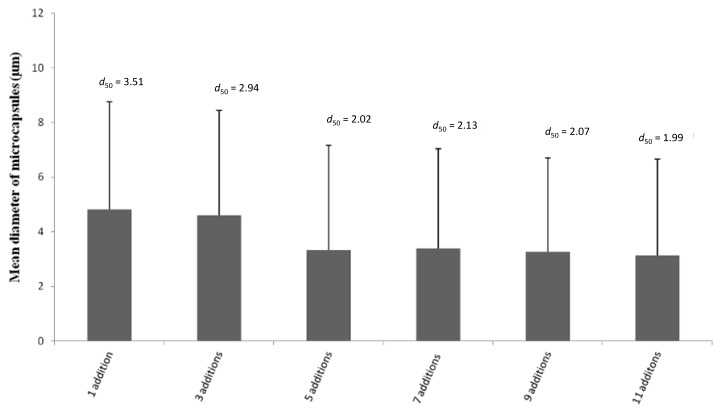
Effect of Chi addition on mean diameter of microcapsules.

The size distribution analysis of microcapsules suspension was performed after addition of Chi during microcapsules formation and it was found that mean diameter of the particles decreased with increasing Chi additions in the system up to five additions. The measurement of mean diameter of microcapsules was made considering diameter range of microcapsules from 0.6 to 30.0 µm. As shown in Figure 3, the microcapsules formed with one addition showed mean diameter of 4.8 ± 3.9 μm and it decreased to 3.2 ± 3.5 μm after five additions, and stayed constant thereafter, indicating that increasing Chi addition during stepwise formation of microcapsules caused strong electrostatic interaction between SDS and Chi on the microcapsules and that led to reduction in the mean diameter of microcapsules by tight packing of layers on the microcapsules [41]. Moreover, the value of *d*_50_ suggested the percentage (%) of particles having a mean diameter of less than 3.5 and 2.0 μm, for 1 and 11 additions, respectively. Thereby, multiple additions of Chi caused tight packing of wall materials on the surface of microcapsules in the study. Moreover, the decrease in mean diameter of the microcapsules with an increase in the number of Chi additions suggests that the coalescence of the particles is likely to be reduced due to strong electrostatic repulsion of positively charged amine groups of Chi present on the surface of the microcapsules. The mean diameter of microcapsules is found to decrease with increasing the number of additions of Chi and, moreover, carrier molecules having size around 1 μm are considered suitable for various applications, especially for drug delivery.

### 3.2. Effect of Plasma Treatment on Wash Durability of Microcapsules Fixed on Textile Substrate (PET Fabric) by Pad-Dry Process

**Figure 4:**
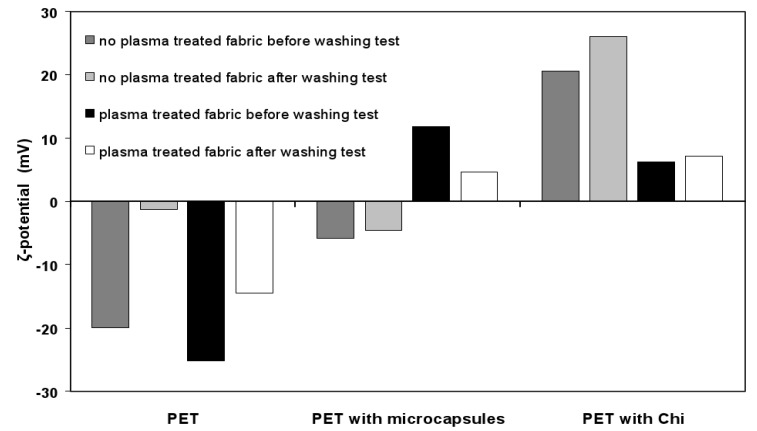
Zeta potential values of PET fabric samples according to the finishing treatment.

In the microcapsules suspension, Chi has positively charged basic amine groups which can effectively interact with acidic carboxyl groups on the surface of PET fabric. The negative zeta potential values of PET fabrics with and without plasma pre-treatment (Figure 4 and Table 1) depicted the presence of similar acidic carboxylic groups at their surfaces [42]. The more negative the zeta potential is, the higher is the density of carboxyl groups at the fiber surface. The atmospheric air plasma-treated PET fabrics have more carboxyl groups at the surface. Indeed, plasma treatments generate polymer chain scissions of the weakest bonds of the PET. These chains of scissions create a large amount of very reactive chain-ends, such as free radicals, which then react easily with the reactive species present in the plasma. These reactions create some oxidized groups like carbonyl, carboxyl and hydroxyl groups at the chain-end.

**Table 1:** Chemical composition of various PET fabric samples before and after washing obtained by analysis of C1s spectrum.

Bonds	PET fabric				PET fabric with microcapsules		PET fabric with Chi	
	No plasma treated		Plasma treated		Plasma treated		Plasma treated	
	Before washing	After washing	Before washing	After washing	Before washing	After washing	Before washing	After washing
–C–C– (%)	72.7	78.9	66.1	76.4	77	77	53.4	53.1
–C–O– (%)	17.3	12.1	20.5	13.6	–	–	–	–
–O–C=O (%)	10	9	13.3	10	–	–	–	–
–C–O–/–C–N– (%)	–	–	–	–	14.4	13.1	33.8	35.5
–O–C=O/–N–C=O (%)	–	–	–	–	8.6	9	12.8	11.4

**Figure 5:**
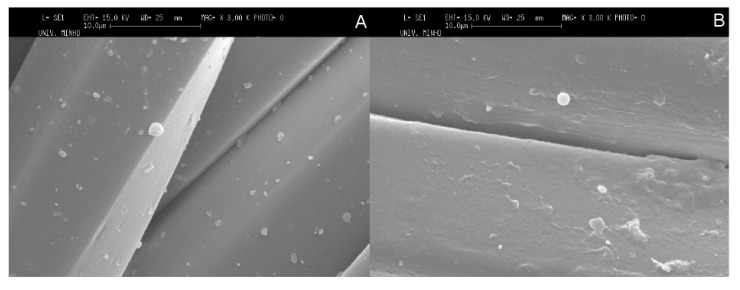
SEM micrographs of polyester (PET) fabrics containing microcapsules without (**A**) and with (**B**) plasma treatment.

The application of washing cycles results in the increase in value of zeta potential of PET fabric samples with and without plasma treatment, and it attributes not only to the elimination of spin finishing oil deposited on the fiber surface during the melt spinning process, but also to the modification of the chemical groups generated during the plasma treatment. Spin finishing oil provides corrective antistatic lubricant that ensures adequate frictional balance between fiber to fiber, and fiber to metal, and reduces static electricity and yellowing. Spin finishing oil provides thermal stability, microfiber cohesion and antistatic protection to fibers. XPS analyses of PET fabric samples with and without plasma treatment showed presence of various carbon groups like –C–C–, –C–O–, and –O–C=O. The percentage of –C–C– group was reduced in PET fabric after plasma treatment, whereas percentage of carbon groups like –C–O– and –O–C=O was increased after plasma treatment of fabric samples. Plasma treatment creates mono-oxidized carbons (C–O–), bi-oxidised carbons (–C=O or –O–C–O–) and tri-oxidised carbons (–O–(C=O)–). The zeta potential of plasma treated PET fabric also indicated that carboxylic groups were introduced after plasma treatment [40]. The introduction of microcapsules onto the surface of fabric increased the zeta potential value up to −5.82 mV, and the value was found close to one after washing. The stable zeta potential with washing cycles shows a good adhesion of microcapsules to the PET fiber. Moreover, zeta potential value of PET fabric surface (−5.82 mV) is the resultant value of the fabric without microcapsules and the same covered with microcapsules. Thereby, it clearly suggests that non-homogeneous deposition or coating of microcapsules occurred onto the surface of PET fabric and it was also supported by SEM micrographs (Figure 5). The plasma treatment of PET fabric enhances its interaction with microcapsules as obtained from their zeta potential values +12.0 and +7.15 mV before and after washing, respectively. PET fabric after pre-activation with plasma treatment allows microcapsules to be deposited more homogenously on surface than that of without plasma pre-activation. The plasma treated PET fabric after coating with microcapsules or Chi solution showed some other groups like –C–N– and –N–C=O in addition to –C–C–, –C–O–, and –O–C=O groups, and these additional groups indicated the presence of amine (–NH_2_) groups of Chi in microcapsules or Chi solution on the surface of PET fabric (Table 1). The zeta potential values of PET fabric coated with Chi before and after washing are higher than other fabric samples, indicating that Chi film has tremendous capacity to interact with PET fabric. This observation also suggested that positively charged amine (–NH_3_^+^) groups of Chi may interact with acidic carboxylic groups of PET substrate by ionic interactions.

Figure 5 shows the SEM images of untreated and plasma treated PET fabrics with microcapsules. On the one hand, untreated PET fabric surface is relatively smooth with some particles (microcapsules) onto it indicating that there is no damage introduced to the surface (Figure 5A). On the other hand, the plasma treatment presents some changes such as blisters and hills to PET fabric on its surface. The blisters on the fabric surface may be resulted from the amorphization of the crystalline domains of PET, involving volume differences at surface and interface of crystalline structure of fabric [43]. The blisters are also likely to be issued from aggregation of microcapsules at the surface of fabric (Figure 5B).

**Figure 6:**
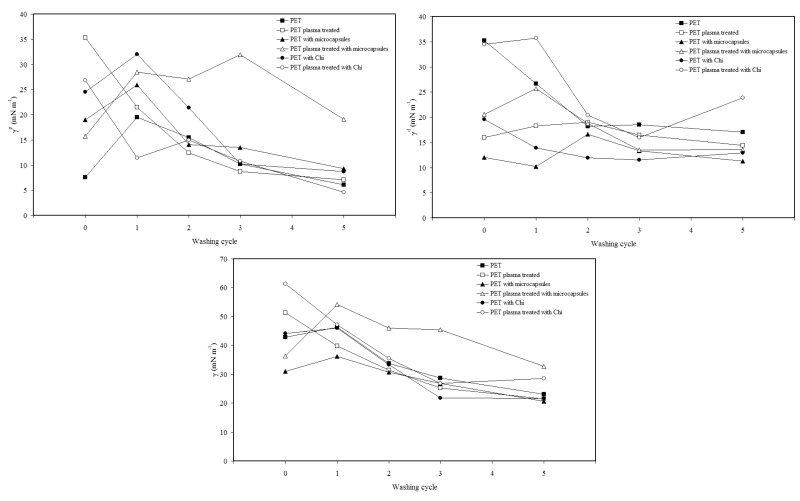
Effect of washing cycles on surface energy components.

The wettability experiments indicated that PET fabric without plasma treatment showed hydrophobic behavior as its γ_s_^d^ value was much higher than γ_s_^p^ (Figure 6). The plasma treatment influences the wettability of PET fabric, and it shows more hydrophilic character after plasma treatment and remains same after up to two washing cycles (Figure 6). However, the surface energy (γ_s_) values of all PET fabric samples increased after plasma treatment. In contrast, the γ_s_^p^ value of PET fabric coated with microcapsules is reduced after plasma treatment indicating that plasma pre-activation caused some rearrangements of polar groups of microcapsules on the surface of PET fabric. During washing, the γ_s_^p^ value of plasma treated fabric containing microcapsules remained the same but other PET fabric samples showed a decrease in the γ_s_^p^ values with the number of washing cycles. This anomaly is mainly due to the rearrangement of polar groups of microcapsules on the surface due to plasma pre-treatment.

**Figure 7:**
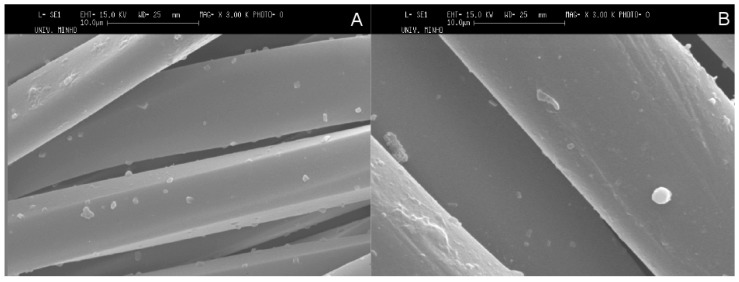
SEM micrographs of untreated (**A**) and treated (**B**) plasma fabrics with microcapsules after washing.

The SEM micrographs of PET fabrics (Figure 7) show the presence of microcapsules on untreated and plasma treated PET fabrics after five washing cycles. Thereby, Chi based microcapsules developed by phase coacervation method based on ionic interactions in the study showed good characteristics of wash durability on the textile substrate even without plasma treatment as pre-activation of substrate. The good wash durability of Chi based microcapsules onto PET fabric samples was considered to be developed by the interaction between positively charged amine groups of Chi in microcapsules and acidic carboxyl groups of PET fabric. In this context, plasma treatment of PET fabric is required to increase the homogenous distribution of microcapsules on the surface of PET fabric and also, the surface energy of the PET fabric samples are increased after plasma treatment. Thereby, plasma treatment of PET fabric increases the wettability of microcapsule sample and this in turn improves the adhesive properties. Thereby, the novelty of our work lies in the fact that we have developed microcapsules which are durable on PET fabric after several washing cycles and the microcapsules sample does not need any prior plasma treatment of PET fabric in order to be durable on the fabric.

## 4. Conclusions

The present study dealt with the development of microcapsules by multilayer phase coacervation method based on ionic interactions between oppositely charged Chi and SDS as wall materials. The microencapsulation method started with the preparation of oil in water emulsion using SDS as an anionic emulsifier. The rinsing step during microcapsule formation was applied to reduce the repulsion between positively charged microcapsules and free Chi macromolecules during microencapsulation process and it reduced the viscosity of the microcapsules suspension. The microcapsules formed after 11 alternate additions of Chi and SDS by phase coacervation process were treated with alkali for drying in liquid medium to solidify the outermost shell of microcapsules. Different characterization techniques like zeta potential, AFM, wetting experiments and surface roughness were applied to observe the change in morphology of microcapsules during step by step synthesis of microcapsules. The alkali treated “11 additions” microcapsules sample was applied on PET fabric by padding process to investigate their wash durability. Additionally, fabric samples were subject to air atmospheric plasma treatment to modify the surface properties of fabric to increase the interaction with the microcapsules. The results obtained from different characterization techniques like wettability experiments, zeta potential and SEM indicated that wash durability of microcapsules on PET fabric was achieved without plasma treatment as preactivation of surface due to unique characteristics of microcapsules to interact with PET substrate. The microcapsules were found to stay on the surface of textile substrate after several washing cycles. The interaction of basic amine groups of Chi in microcapsules with acidic carboxylic groups of PET fabric with and without plasma treatment led to wash-durable microcapsules on PET fabric.
